# Editorial: Environmental microbiomes, metabolites, and respiratory diseases

**DOI:** 10.3389/frmbi.2024.1388525

**Published:** 2024-03-13

**Authors:** Xi Fu, Dan Norbäck, Yu Sun

**Affiliations:** ^1^ Guangdong Provincial Engineering Research Center of Public Health Detection and Assessment, School of Public Health, Guangdong Pharmaceutical University, Guangzhou, China; ^2^ Occupational and Environmental Medicine, Department of Medical Science, University Hospital, Uppsala University, Uppsala, Sweden; ^3^ Guangdong Provincial Key Laboratory of Protein Function and Regulation in Agricultural Organisms, College of Life Sciences, South China Agricultural University, Guangdong, Guangzhou, China

**Keywords:** indoor microbiome, respiratory diseases, nasopharyngeal microbiome, next-generation sequencing, microbial community succession

In this Research Topic of Frontiers in Microbiomes, we delve into the intricate connections between environmental microbiomes and respiratory diseases. Through a collection of cutting-edge research, reviews, and perspectives, we illuminate the roles of environmental microbial communities in influencing respiratory health. The diversity of environmental microbiomes, from the air we breathe and the surfaces within our homes, creates a complex web of interactions with the human body, particularly affecting the respiratory system.

The articles in this Research Topic address a range of pertinent topics, from the composition and diversity of environmental and respiratory microbiomes to the mechanisms by which these microbiomes affect the immune system and contribute to respiratory diseases like asthma, rhinitis, and infections. Our contributors have utilized advanced methodologies, from next-generation sequencing to innovative computational approaches, to explore these interactions in depth.

In the study by McDaneld et al., a detailed investigation into the outbreak of bovine respiratory disease (BRD) among nursing beef calves is presented. The research meticulously characterizes both bacterial and viral populations within the upper respiratory tract at two critical time points: initial vaccination and the BRD outbreak. By employing advanced DNA and RNA extraction techniques followed by next-generation sequencing, the study uncovers a significant presence of bovine coronavirus and Mycoplasma bovirhinis during the outbreak, not previously detected at high abundance. This pivotal work not only enhances our understanding of microbial dynamics in relation to BRD but also underscores the complex interplay of commensal bacteria and viral pathogens in animal health.

Zimmermann’s systematic review delves into the nasopharyngeal microbiome of children, emphasizing its dynamic nature and crucial role in respiratory health and immune system development. By synthesizing findings from numerous studies, Zimmermann identifies key microbial profiles associated with various respiratory conditions, such as infections and asthma exacerbations. The review highlights how external factors, including birth mode, breastfeeding status, and antibiotic use, influence the nasopharyngeal microbiome, pointing to its potential as both a marker for disease risk and a target for preventive strategies.

Fu et al.’s research explores the impact of the indoor microbiome on rhinitis symptoms among university students, providing novel insights into how environmental characteristics influence respiratory health. Through the analysis of settled air dust microbiome in dormitory rooms and comprehensive surveys, the study establishes a correlation between specific microbial genera and rhinitis symptoms. The findings suggest that manipulating indoor microbiome composition could serve as an innovative approach to preventing or mitigating rhinitis, highlighting the importance of environmental factors in respiratory disease prevalence.

Liu et al. investigate the microbial community succession during the composting of cow manure and tobacco straw, offering valuable perspectives on the environmental microbiome’s impact on respiratory health. Through amplicon sequencing and shotgun metagenomics, the study reveals the dominant microbial genera present and tracks the temporal dynamics of the microbial community. This research not only contributes to our understanding of the composting process but also raises awareness of the potential respiratory health implications associated with exposure to microbial communities in agricultural settings.

Each of these contributions within this Research Topic underscores the profound effect environmental microbiomes have on respiratory health, paving the way for future research and interventions aimed at reducing the incidence and severity of respiratory diseases.

## Author contributions

XF: Writing – original draft, Writing – review & editing. DN: Writing – review & editing. YS: Writing – original draft, Writing – review & editing.

